# Mechanical Performance of Milled CAD/CAM Versus 3D-Printed Dental Prostheses: A Systematic Review and Meta-Analysis of Flexural Strength and Fracture Resistance

**DOI:** 10.3390/dj14060325

**Published:** 2026-05-29

**Authors:** Luis Chauca-Bajaña, Gabriela Guadalupe Zambrano Manzaba, Andrea Ordoñez-Balladares, Rosangela Caicedo-Quiroz, Marcos Daniel Rodríguez Zuleta, Juan Carlos Suarez Palacios, Nayely Teran-Sánchez, Andrea Carolina Sánchez Salcedo, Byron Velasquez Ron

**Affiliations:** 1 School of Dentistry, Universidad Católica de Santiago de Guayaquil (UCSG), Guayas 090101, Ecuador; luis.chaucab@ug.edu.ec (L.C.-B.); gabriela.zambrano06@cu.ucsg.edu.ec (G.G.Z.M.); 2 School of Dentistry, Universidad Bolivariana del Ecuador, Durán 092406, Ecuador; 3 Oral Rehabilitation, College Dentistry, University of Guayaquil, Guayaquil 090101, Ecuadorandrea.sanchezs@ug.edu.ec (A.C.S.S.); 4 Carrera de Odontología, Universidad de Las Américas Ecuador (UDLA), Quito 170102, Ecuador; byron.velasquez@udla.edu.ec

**Keywords:** computer-aided design, dental prosthesis, flexural strength, fracture resistance

## Abstract

**Background/Objectives:** The growing adoption of digital technologies in prosthodontics has led to the widespread use of computer-aided design and computer-aided manufacturing (CAD/CAM) and three-dimensional (3D) printing for dental prostheses. However, differences in mechanical performance, particularly flexural strength and fracture resistance, remain a concern. Objective: To systematically evaluate and compare the flexural strength and fracture resistance of milled CAD/CAM and 3D-printed dental prostheses. **Methods:** A systematic review and meta-analysis were conducted following PRISMA 2020 guidelines. A comprehensive search was performed across multiple databases, including PubMed, Scopus, Web of Science, and Cochrane Library. In vitro studies comparing milled and 3D-printed prosthetic materials were included. Data extraction and risk of bias assessment were performed independently by multiple reviewers. A random-effects meta-analysis using standardized mean differences (SMD) was conducted. **Results:** Five studies were included in the meta-analysis for flexural strength. Milled CAD/CAM materials demonstrated significantly higher flexural strength compared to 3D-printed resins (SMD = 3.70; 95% CI: 0.80–6.59; *p* = 0.012), with substantial heterogeneity (I^2^ = 93.3%). Fracture resistance results were inconsistent and influenced by individual studies, with sensitivity analyses showing variability in pooled estimates. Overall, the risk of bias was considered low, although some concerns were identified in randomization and blinding. **Conclusions:** CAD/CAM-milled materials exhibit superior flexural strength, while fracture resistance outcomes remain variable. Although 3D-printed materials may be clinically acceptable, further standardized studies are required to confirm their mechanical reliability.

## 1. Introduction

The rapid integration of digital technologies into prosthodontics has profoundly changed the way dental prostheses are designed and manufactured. In particular, computer-aided design and computer-aided manufacturing (CAD/CAM) and additive manufacturing (3D printing) have emerged as dominant approaches, offering improved standardization, reduced chairside time, and enhanced reproducibility compared to conventional techniques [1,2,3]. These advancements have expanded the range of materials and fabrication workflows available for both provisional and definitive restorations.

Despite these advantages, the clinical success of dental prostheses continues to depend largely on their mechanical performance, especially flexural strength and fracture resistance. Flexural strength reflects the ability of a material to resist deformation under bending forces, while fracture resistance represents its capacity to withstand catastrophic failure under functional loading [4,5,6]. These properties are particularly critical in posterior regions, where restorations are subjected to higher occlusal stresses and cyclic fatigue.

CAD/CAM-milled materials are typically fabricated from pre-polymerized industrial blocks under optimized conditions of pressure and temperature, resulting in a highly homogeneous microstructure with minimal internal defects and superior mechanical properties [7,8,9]. In contrast, 3D printing technologies fabricate restorations layer by layer, which, although advantageous in terms of material efficiency and the ability to produce complex geometries, may introduce anisotropy, interlayer bonding weaknesses, and increased porosity [10,11,12].

A growing number of in vitro studies have attempted to compare the mechanical behavior of milled and 3D-printed dental materials. Several investigations have reported that CAD/CAM-milled resins exhibit significantly higher flexural strength and fracture resistance compared to their 3D-printed counterparts [13,14,15]. However, other studies suggest that under specific conditions—such as optimized printing orientation, adequate post-curing protocols, and improved resin formulations—3D-printed materials may achieve comparable mechanical performance [16,17,18].

Furthermore, variability in testing methodologies, material compositions, and fabrication parameters has contributed to inconsistent findings across studies, limiting the generalizability of the available evidence [19]. Recent systematic reviews have attempted to synthesize these data; nevertheless, many have focused on specific materials or isolated mechanical properties, without providing a comprehensive comparison of flexural strength and fracture resistance across different fabrication technologies [20].

Given the continuous evolution of both CAD/CAM and additive manufacturing systems, as well as the increasing clinical use of 3D-printed prostheses, there is a clear need for an updated and comprehensive evaluation of their mechanical performance. Therefore, the aim of this systematic review and meta-analysis is to compare the flexural strength and fracture resistance of milled CAD/CAM and 3D-printed dental prostheses, providing clinically relevant evidence to guide material selection and improve long-term treatment outcomes.

## 2. Materials and Methods

**Figure 1:**
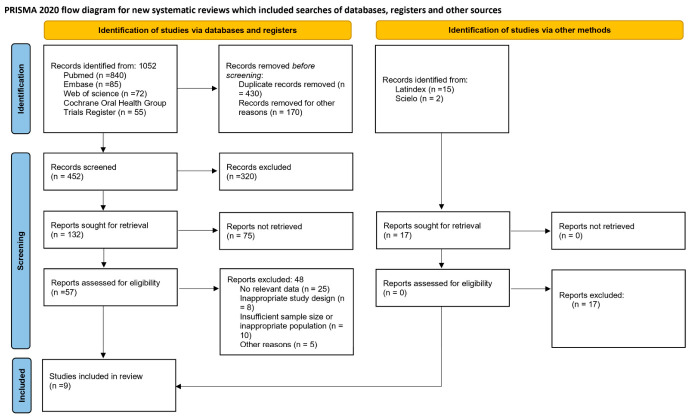
Flowchart of selected studies.

A comprehensive search of the PROSPERO database was conducted in January 2026 to ensure that no similar ongoing reviews were registered. Subsequently, the protocol of this systematic review and meta-analysis was prospectively registered under the identification number CRD420261368405. The study was designed, conducted, and reported in accordance with the PRISMA 2020 statement, and the corresponding flow diagram is presented in Figure 1 [21]. In addition, the completed PRISMA 2020 checklist is provided as Supplementary Material (File S1).

### 2.1. PICO Question

Among dental prostheses fabricated using digital manufacturing technologies, is there a significant difference in flexural strength and fracture resistance between milled CAD/CAM and 3D-printed materials?

PICO Framework

Population (P): Dental prostheses fabricated for provisional or definitive restorations, including standardized laboratory specimens and prosthetic restorations designed for mechanical testing under controlled experimental conditions.

Although differences existed between specimen-based and prosthesis-based models, all included studies evaluated comparable mechanical outcomes related to flexural strength or fracture resistance and directly compared CAD/CAM-milled versus 3D-printed materials.

Intervention (I): Milled CAD/CAM dental prostheses (subtractive manufacturing).

Comparison (C): 3D-printed dental prostheses (additive manufacturing).

Outcomes (O):

- Primary outcomes:
  ○ Flexural strength (MPa)
  ○ Fracture resistance (N)
- Secondary outcomes (optional, if the study decides to expand later):
  ○ Elastic modulus
  ○ Weibull modulus
  ○ Failure mode análisis

### 2.2. Search Strategy and Database Screening

The study selection process was conducted using the Rayyan platform (Qatar Computing Research Institute, Doha, Qatar), which facilitated the identification, organization, and blinded screening of the retrieved records.

A comprehensive and systematic literature search was carried out across multiple electronic databases, including MEDLINE via PubMed, EMBASE via OVID, Web of Science, Scopus, the Cochrane Library, and ClinicalTrials.gov. Additionally, the five regional databases of the World Health Organization (AIM, LILACS, IMEMR, IMSEAR, and WPRIM) were explored to ensure broad coverage of the available evidence. To minimize publication bias and identify grey literature, conference proceedings were also screened through the Conference Proceedings Citation Index.

The search strategy was customized for each database using a combination of controlled vocabulary terms (e.g., MeSH terms) and free-text keywords related to digital dental manufacturing and mechanical properties. The main search terms included “CAD/CAM”, “computer-aided design”, “computer-aided manufacturing”, “3D printing”, “additive manufacturing”, “dental prosthesis”, “provisional restorations”, “flexural strength”, “fracture resistance”, “mechanical properties”, and “dental materials”. Boolean operators (AND/OR) were systematically applied to combine search terms and enhance both sensitivity and specificity.

To ensure completeness, a manual search of the reference lists of relevant studies and previously published reviews was performed to identify additional eligible articles that may not have been retrieved through the electronic search. All records identified were imported into the Rayyan platform, where duplicate entries were detected and removed prior to the screening process. Subsequently, titles and abstracts were independently assessed to determine their eligibility according to the predefined inclusion and exclusion criteria.

### 2.3. Eligibility Criteria

Inclusion Criteria:

Study design: In vitro experimental studies; controlled laboratory studies; randomized in vitro comparisons; clinical studies (if fracture resistance evaluated mechanically). 2. Population/material: Dental prostheses (crowns, bridges, provisional FDPs, fixed restorations); specimens fabricated specifically for prosthetic application. 3. Intervention: CAD/CAM milled resin-based materials; industrial or chairside milled blocks. 4. Comparison: 3D-printed resin prostheses (SLA, DLP, LCD, etc.). 5. Outcomes: Flexural strength measured via three-point bending test; four-point bending test; ISO 10477 or ISO 4049 protocols; fracture resistance measured in Newtons (N). 6. Data reporting: Mean and standard deviation available; sample size clearly reported; units clearly specified. 7. Language: English. 8. Publication type: Peer-reviewed original research articles.

Exclusion Criteria:

Studies evaluating: Only hardness; only wear; only surface roughness; only bond strength (unless fracture resistance also included). 2. Studies without: Direct comparison between milled and 3D-printed groups; quantitative mechanical data. 3. Case reports. 4. Narrative reviews. 5. Systematic reviews. 6. Finite element analysis studies. 7. Studies on: Denture bases only (unless fracture resistance evaluated); orthodontic appliances; surgical guides. 8. Studies combining materials without separate subgroup data.

Although variability existed regarding resin composition, prosthesis type, and testing protocols, studies were considered eligible when they evaluated comparable mechanical outcomes (flexural strength or fracture resistance) under standardized laboratory conditions and directly compared CAD/CAM-milled versus 3D-printed materials. To account for expected methodological and material heterogeneity, a random-effects model using standardized mean differences (SMD) was applied.

### 2.4. Study Screening and Data Extraction

A standardized data extraction form was specifically developed for this review and independently completed by three investigators (LC, AOB, and BVR) to ensure consistency and reproducibility. Any discrepancies or uncertainties during the extraction process were resolved through discussion and consensus with three additional investigators (JS, RC, and MR), who were blinded to the study objectives.

For each eligible study, the following variables were systematically extracted: first author and year of publication, country of origin, study design (in vitro experimental or comparative), type of CAD/CAM milled material, type of 3D-printed material, sample size per group, mechanical property evaluated (e.g., flexural strength or fracture resistance), testing method (e.g., three-point bending test or universal testing machine), and the main findings reported by the authors. When multiple mechanical outcomes were assessed within the same study, only those relevant to the predefined outcomes of interest were included in the analysis.

All extracted data were compiled and organized in a structured table format (Table 1) to facilitate comparison across studies. Particular attention was given to ensuring consistency in the classification of materials and testing methods to allow for appropriate qualitative synthesis and subsequent quantitative analysis.

To further enhance the interpretation of the included studies, an additional qualitative synthesis was performed, summarizing key methodological characteristics and experimental conditions across studies (Table 2). This table includes detailed information regarding study design, materials evaluated, sample size, testing protocols, main outcomes, and relevant observations. This complementary qualitative assessment allowed a more comprehensive comparison of the included studies and facilitated the identification of methodological variability, which was considered during data interpretation and synthesis.

### 2.5. Assessment of Risk of Bias (RoB)

The methodological quality and risk of bias of the included studies were independently assessed by three reviewers using a domain-based approach adapted for in vitro studies. The following domains were evaluated: specimen preparation (D1), randomization (D2), blinding (D3), outcome measurement (D4), and statistical analysis/reporting. Each domain was classified as “low risk of bias” or “some concerns” according to predefined criteria [31].

Disagreements among reviewers were resolved through discussion and, when necessary, by consultation with additional investigators until consensus was achieved. The results of the risk of bias assessment were graphically represented using both a traffic light plot and a summary bar chart to facilitate visualization and interpretation of methodological quality across studies [32].

### 2.6. Statistical Analysis

A quantitative synthesis was performed to compare the mechanical performance between CAD/CAM-milled and 3D-printed dental prostheses. Standardized mean differences (SMD) with corresponding 95% confidence intervals (CI) were calculated for each study due to variability in measurement scales and testing protocols. The use of SMD allowed the pooling of studies with differences in material composition, prosthesis design, specimen dimensions, and testing methodologies by standardizing effect sizes across heterogeneous experimental conditions. Although methodological and material variability was expected among the included in vitro studies, all studies evaluated comparable mechanical outcomes and directly compared CAD/CAM-milled versus 3D-printed prosthetic materials under controlled laboratory conditions. A random-effects model using restricted maximum likelihood (REML) was applied to account for between-study heterogeneity. Statistical heterogeneity was assessed using Cochran’s Q test and quantified with the I^2^ statistic, with values above 50% indicating substantial heterogeneity. Prediction intervals were additionally calculated to estimate the expected range of effects in future studies. Publication bias and small-study effects were explored through visual inspection of funnel plots. Sensitivity analyses were conducted using a leave-one-out approach to evaluate the robustness of the pooled estimates. Furthermore, influence diagnostics, including Baujat plots and influence analysis (studentized residuals, DFFITS, and Cook’s distance), were performed to identify studies contributing to heterogeneity and overall effect size. All statistical analyses were conducted using R software 4.4.1 (R Foundation for Statistical Computing, Vienna, Austria).

## 3. Results

### 3.1. Flexural Strength

**Figure 2:**
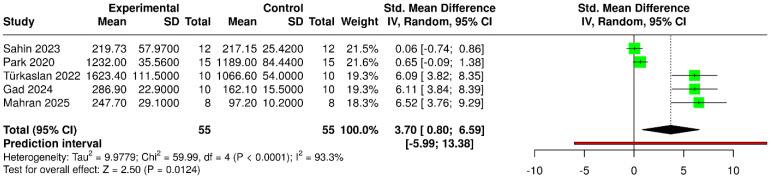
Forest plot of the meta-analysis of flexural strength [22,23,24,25,26].

The meta-analysis of five in vitro studies showed that CAD/CAM-milled resins exhibited significantly higher flexural strength than 3D-printed resins (SMD = 3.70; 95% CI: 0.80–6.59; *p* = 0.012). However, substantial heterogeneity was observed (I^2^ = 93.3%), indicating considerable variability among studies. Most studies favored milled materials, although one study reported no significant difference. The wide prediction interval (−5.99 to 13.38) suggests that the effect may vary depending on material type and testing conditions (Figure 2).

**Figure 3:**
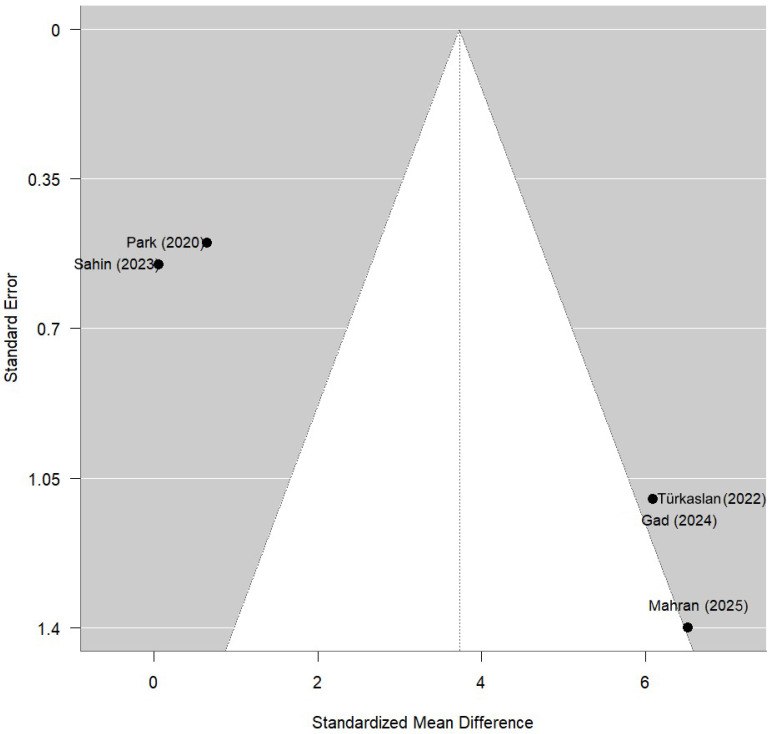
Funnel plot of the meta-analysis of flexural strength [22,23,24,25,26].

The funnel plot showed an asymmetric distribution of the included studies. Studies with larger effect sizes (e.g., Türkaslan 2022 [24], Gad 2024 [25], and Mahran 2025 [26]) were located on the right side of the plot, while those with smaller or non-significant effects (Sahin 2023 [22] and Park 2020 [23]) clustered near the line of no effect. This pattern may suggest the presence of publication bias or small-study effects; however, given the limited number of included studies, this finding should be interpreted with caution (Figure 3).

**Figure 4:**
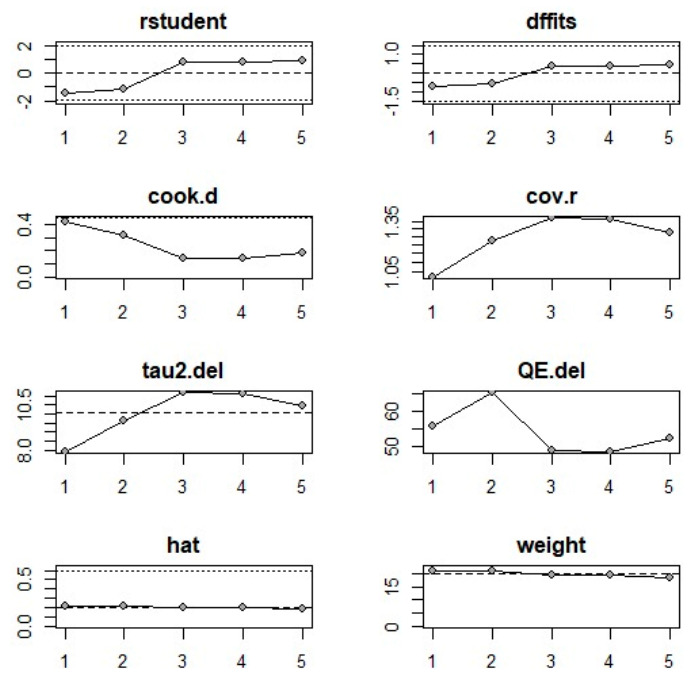
Influence diagnostics of the meta-analysis of flexural strength.

Influence diagnostics indicated that no single study exerted a disproportionate influence on the pooled effect size. Studentized residuals and DFFITS values remained within acceptable limits, suggesting the absence of outliers, while Cook’s distance values were low across all studies. However, tau^2^ and QE diagnostics suggested that Türkaslan (2022) [24] and Gad (2024) [25] contributed more substantially to between-study heterogeneity. Overall, the results indicate that the meta-analysis was not driven by any single study, although some studies had a greater impact on heterogeneity (Figure 4).

**Figure 5:**
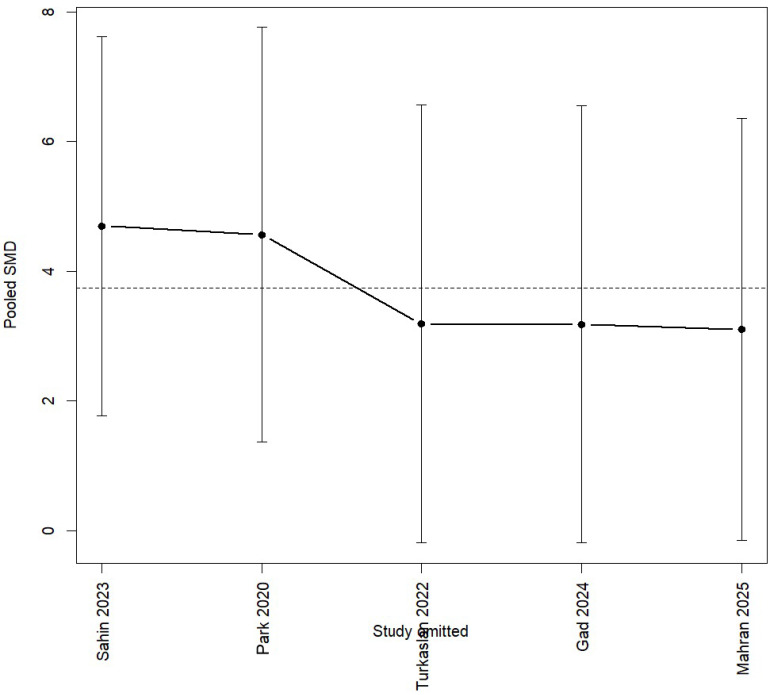
Leave-one-out sensitivity analysis of the meta-analysis of flexural strength [22,23,24,25,26].

Leave-one-out sensitivity analysis showed that the pooled effect size remained relatively stable after sequential exclusion of individual studies. However, the omission of Türkaslan (2022) [24], Gad (2024) [25], and Mahran (2025) [26] resulted in a reduction in the pooled standardized mean difference. Despite this decrease, the overall direction of the effect remained unchanged, consistently favoring CAD/CAM-milled materials. These findings indicate that the results are robust, although certain studies contributed more strongly to the magnitude of the effect (Figure 5).

### 3.2. Risk of Bias—Traffic Light Plot

**Figure 6:**
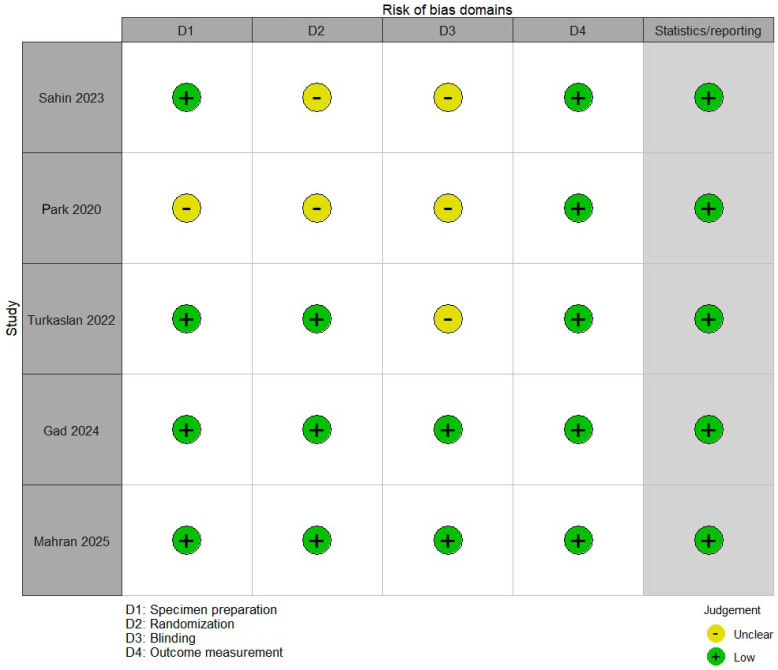
Risk of bias assessment of included studies (traffic light plot) [22,23,24,25,26].

The risk of bias assessment indicated that most studies presented a low risk of bias across the evaluated domains. Outcome measurement and statistical reporting were consistently rated as low risk in all studies. Some concerns were identified mainly in the domains of randomization and blinding, particularly in Sahin (2023) [22] and Park (2020) [23], where these aspects were unclear. Overall, the methodological quality of the included studies was considered acceptable (Figure 6).

**Figure 7:**
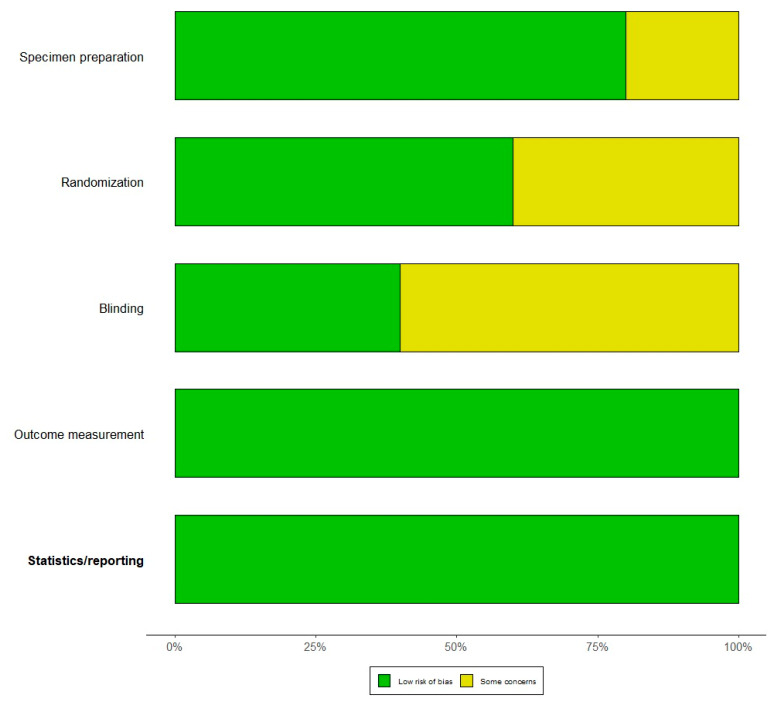
Risk of bias summary of included studies.

The risk of bias summary showed that most domains were classified as low risk across the included studies. Outcome measurement and statistical reporting demonstrated consistently low risk, while specimen preparation showed predominantly low risk with minor concerns. In contrast, randomization and especially blinding presented a higher proportion of studies with some concerns. Overall, these findings indicate acceptable methodological quality, although limitations remain in study design aspects such as allocation and blinding (Figure 7).

### 3.3. Fracture Resistance

**Figure 8:**
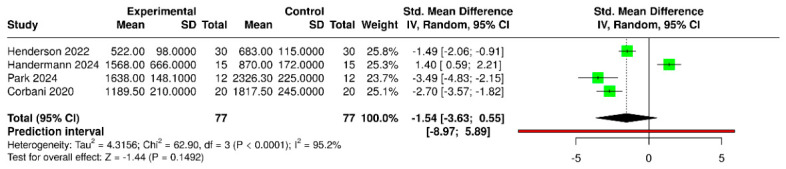
Forest plot comparing fracture resistance between CAD/CAM-milled and 3D-printed resins [27,28,29,30].

The meta-analysis of four in vitro studies showed no statistically significant difference in fracture resistance between CAD/CAM-milled and 3D-printed resins (SMD = −1.54; 95% CI: −3.63 to 0.55; *p* = 0.149). However, substantial heterogeneity was observed (I^2^ = 95.2%), indicating considerable variability among the included studies. While some studies favored CAD/CAM-milled materials, others reported higher fracture resistance in 3D-printed resins, contributing to the inconsistency of the pooled results. The wide prediction interval (−8.97 to 5.89) further suggests that the true effect may vary considerably depending on material composition and experimental conditions (Figure 8).

**Figure 9:**
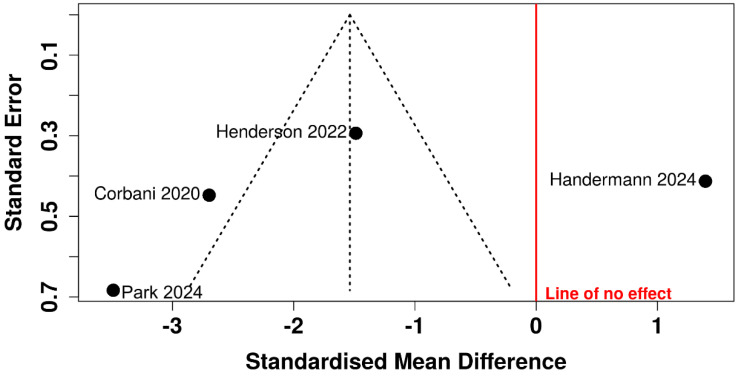
Funnel plot of the meta-analysis of fracture resistance [27,28,29,30].

The funnel plot showed an asymmetric distribution of the included studies. Most studies were located on the left side of the plot, indicating a tendency toward higher fracture resistance in CAD/CAM-milled materials, whereas Handermann (2024) [29] showed an opposite effect, favoring 3D-printed materials. This asymmetry suggests potential publication bias or heterogeneity related to differences in materials and testing conditions. However, given the small number of included studies, this finding should be interpreted with caution (Figure 9).

**Figure 10:**
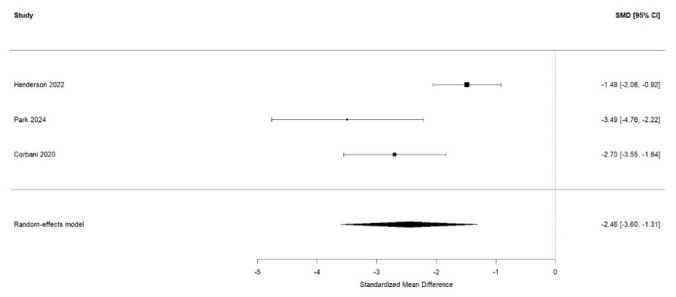
Forest plot of the sensitivity analysis of fracture resistance after exclusion of the influential study [27,28,30].

A sensitivity analysis was performed excluding the study by Handermann (2024) [29], which showed an opposite effect direction compared with the other included studies. After its removal, the pooled analysis demonstrated a statistically significant effect favoring CAD/CAM-milled materials (SMD = −2.46; 95% CI: −3.60 to −1.31). In addition, heterogeneity was notably reduced, indicating that the overall results were strongly influenced by this study. These findings suggest that the variability in fracture resistance outcomes is largely driven by individual study characteristics (Figure 10).

**Figure 11:**
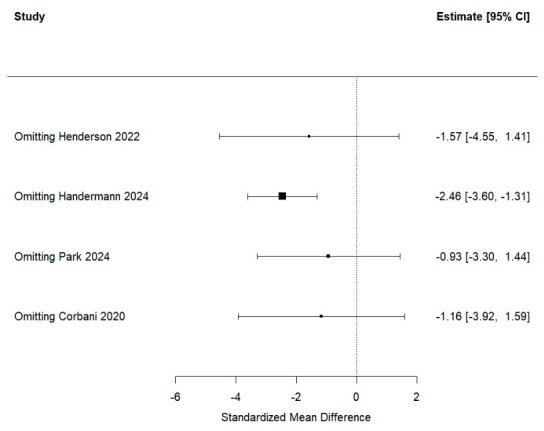
Leave-one-out sensitivity analysis plot of fracture resistance [27,28,29,30].

Leave-one-out sensitivity analysis showed that the pooled effect was highly influenced by the study of Handermann (2024) [29]. Exclusion of this study resulted in a statistically significant effect favoring CAD/CAM-milled materials (SMD = −2.46; 95% CI: −3.60 to −1.31), whereas omission of any other study did not materially change the overall non-significant result. These findings indicate that the overall meta-analysis is not robust and is strongly dependent on a single study (Figure 11).

**Figure 12:**
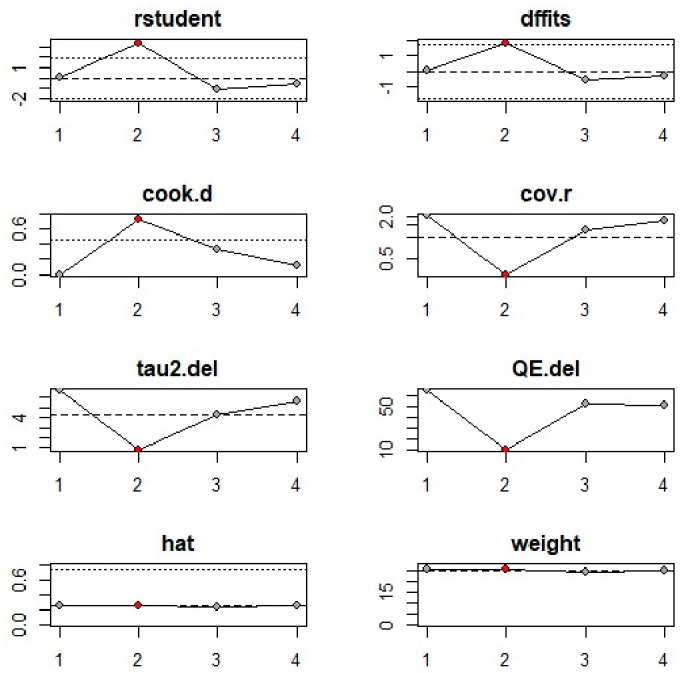
Influence diagnostics plot of fracture resistance.

Influence diagnostics identified Handermann (2024) [29] as the most influential study across multiple metrics, including studentized residuals, DFFITS, and Cook’s distance, where it exceeded the recommended thresholds. In addition, its removal resulted in a substantial reduction in tau^2^ and QE, indicating a marked decrease in between-study heterogeneity. The remaining studies showed minimal influence on the pooled effect. These findings confirm that the overall meta-analysis of fracture resistance is strongly driven by a single study (Figure 12).

**Figure 13:**
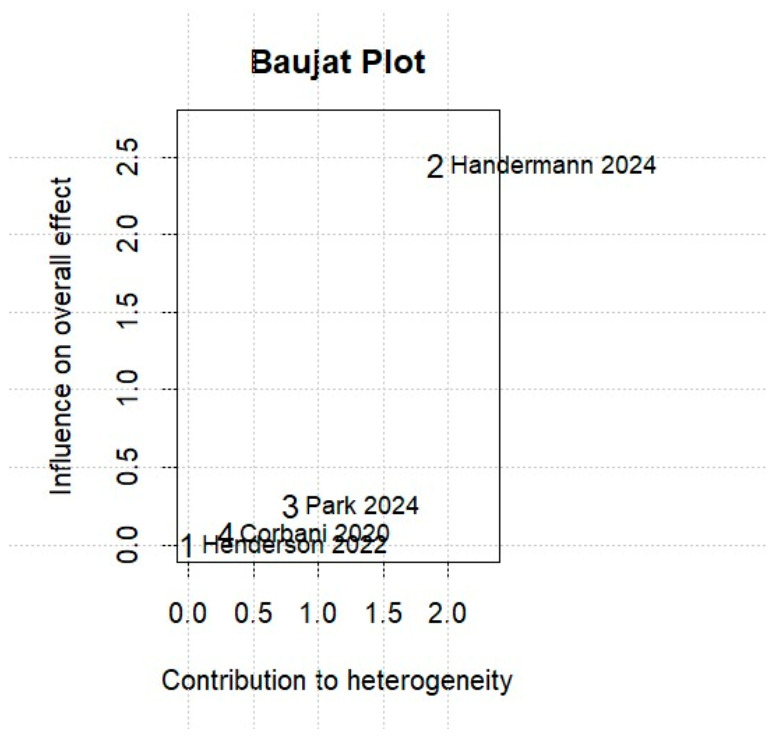
Contribution to heterogeneity [27,28,29,30].

The Baujat plot identified Handermann (2024) [29] as the study with the greatest contribution to both overall heterogeneity and influence on the pooled effect size. In contrast, the remaining studies (Henderson 2022 [28], Park 2024 [30], and Corbani 2020 [27]) showed minimal contribution to heterogeneity and limited influence on the overall estimate. These findings further confirm that the variability and instability observed in the meta-analysis are largely driven by a single influential study (Figure 13).

## 4. Discussion

This systematic review with meta-analysis demonstrated that CAD/CAM-milled materials exhibit significantly higher flexural strength than 3D-printed resins, with a large effect size (SMD = 3.70; 95% CI: 0.80–6.59; *p* = 0.012), indicating a mechanically meaningful difference between the two manufacturing pathways. These findings are consistent with previous evidence suggesting that materials polymerized under highly controlled conditions achieve a higher degree of conversion, which directly translates into improved mechanical properties. In this context, Stansbury emphasized that polymerization efficiency is a critical determinant of the final mechanical performance of resin-based materials, in agreement with prior studies on both printed and conventionally polymerized provisional materials [2,10]. Importantly, this difference should not be interpreted as an intrinsic superiority of CAD/CAM materials per se, but rather as a reflection of the greater technological maturity and process control associated with these systems.

From a microstructural standpoint, the superior performance of CAD/CAM materials can be attributed to a more homogeneous polymer network, reduced internal defects, and a more stable structural organization. In contrast, additive manufacturing produces a layered architecture in which interlaminar interfaces may act as stress concentration sites, facilitating crack initiation and propagation. These inherent limitations of additive processes have been widely described, together with the evidence of increased variability in the flexural strength of 3D-printed resins depending on the manufacturing system and processing conditions [3,23].

The overall heterogeneity observed in the meta-analysis was high (I^2^ = 93.3%), indicating that a substantial proportion of the variability arises from true differences among the included studies rather than random error. Similar heterogeneity was also observed in the fracture resistance analysis. This finding is consistent with the current literature, which highlights that multiple factors—including material composition, printing orientation, aging protocols, and testing methodologies—may influence the mechanical behavior of printed materials [33,34].

Despite this variability, quantitative synthesis was considered appropriate because all included studies directly compared CAD/CAM-milled and 3D-printed prosthetic materials using comparable mechanical outcomes under controlled laboratory conditions. The use of standardized mean differences and a random-effects model allowed estimation of the overall effect while accounting for inter-study heterogeneity. Subgroup analyses were not performed due to the limited number of studies and the diversity of experimental protocols, which could have produced unreliable subgroup estimates. Therefore, the pooled effect size should be interpreted cautiously as an overall trend rather than a definitive quantitative estimate.

Among the key variables in additive manufacturing, printing orientation, layer thickness, and post-curing protocols appear to play a decisive role in determining the final mechanical response. Significant differences in microhardness and strength have been reported depending on the fabrication method, along with superior dimensional accuracy in milled restorations compared with printed counterparts [35,36]. The lack of standardization in these processing parameters remains a major limitation, hindering direct comparison across studies.

Marginal adaptation represents another critical factor influencing stress distribution and overall mechanical performance. Previous studies have shown that digitally fabricated restorations may exhibit variations in marginal fit compared with conventional techniques, and that achieving consistent outcomes in additive manufacturing remains challenging due to the sensitivity of operational parameters [36,37]. In this context, improved geometric accuracy may contribute to a more uniform distribution of functional loads, thereby enhancing clinical performance.

From a materials science perspective, the processing–structure–properties relationship provides a comprehensive framework to interpret these findings. The evolution of dental biomaterials has been closely associated with the advancement of digital technologies, with CAD/CAM systems representing a pivotal development in modern dentistry [38,39]. These systems enable highly controlled and reproducible manufacturing processes, in contrast to the current variability associated with additive techniques.

Clinically, the advantages of CAD/CAM systems have also been reported in implant-supported restorations and more complex prosthetic applications, primarily due to their superior precision, reproducibility, and continuous improvements in manufacturing accuracy and efficiency [40,41]. However, caution is required when extrapolating the present findings to clinical scenarios, as all included studies were conducted under controlled in vitro conditions. Laboratory mechanical testing cannot fully reproduce the complexity of the oral environment, including cyclic fatigue, saliva exposure, thermal fluctuations, pH changes, and patient-specific occlusal dynamics. Therefore, the present results should be interpreted primarily as comparative mechanical evidence rather than direct predictors of long-term clinical performance.

An additional limitation of this review is the relatively small number of studies included in the quantitative synthesis, particularly for fracture resistance. The limited sample of available studies may reduce statistical power and increase the instability of pooled estimates, especially in the presence of substantial heterogeneity. For this reason, the findings should be interpreted cautiously. Nevertheless, complementary analyses, including leave-one-out sensitivity analysis, prediction intervals, and influence diagnostics, were performed to evaluate the robustness and consistency of the results.

Finally, three-dimensional accuracy remains a key determinant of restoration performance. CAD/CAM systems have been shown to achieve high-dimensional precision, which facilitates improved marginal adaptation and more homogeneous stress distribution [42]. Overall, the available evidence suggests that, although 3D printing represents a promising and rapidly evolving technology, CAD/CAM materials currently maintain a clear advantage in terms of flexural strength and mechanical predictability under the conditions evaluated. Future research should prioritize the standardization of additive manufacturing protocols to enhance inter-study comparability and support their translation into clinical practice.

## 5. Conclusions

Milled CAD/CAM materials generally demonstrated superior flexural strength compared to 3D-printed resins, indicating a trend toward more consistent mechanical performance under laboratory conditions. In contrast, fracture resistance results were heterogeneous and strongly influenced by individual studies. Although 3D-printed materials showed lower values in some cases, their mechanical behavior may vary depending on processing conditions and material composition. Given the limited number of available studies and the substantial heterogeneity observed, the findings should be interpreted cautiously. Further standardized in vitro and clinical studies are needed to clarify the long-term mechanical reliability and clinical applicability of these materials.

## Figures and Tables

**Table 1:** Characteristics of the studies included in the systematic review and meta-analysis.

Author (Year)	Country	Study Design	Milled Material	3D-Printed Material	Sample Size (per Group)	Mechanical Property Evaluated	Testing Method	Main Finding
Sahin [22] (2023)	Turkey	In vitro comparative study	CAD/CAM milled resin composite	3D-printed resin composite	12/12	Flexural strength	Three-point bending test	No significant difference between milled and printed groups.
Park [23] (2020)	South Korea	In vitro experimental study	CAD/CAM milled composite resin	3D-printed resin	15/15	Flexural strength	Universal testing machine	Milled materials showed slightly higher flexural strength than printed resins.
Türkaslan [24] (2022)	Turkey	In vitro comparative study	CAD/CAM milled PMMA	3D-printed PMMA resin	10/10	Flexural strength	Three-point bending test	Milled materials exhibited significantly higher flexural strength.
Gad [25] (2024)	Egypt	In vitro experimental study	CAD/CAM milled fixed dental prosthesis resin	3D-printed permanent resin	10/10	Flexural strength	Universal testing machine	Milled prosthetic materials demonstrated superior mechanical performance.
Mahran [26] (2025)	Egypt	In vitro comparative study	CAD/CAM milled provisional resin	3D-printed provisional resin	8/8	Flexural strength	Mechanical testing using universal testing machine	Milled materials showed significantly higher flexural strength than printed resins.

**Table 2:** Qualitative summary of in vitro studies evaluating fracture resistance of CAD/CAM and 3D-printed resin materials.

Authors (Year)	Study Design	Materials Compared	Sample Size	Test Type	Methodology	Main Results	Conclusion
Corbani et al. [27] (2020)	In vitro experimental	3D-printed composite resin vs. CAD/CAM milled composite resin crowns	n = 60 (10 per subgroup; thickness: 0.5, 1.0, 1.5 mm)	Fracture resistance (N)	Thermocycling (5000 cycles, 5–55 °C) + cyclic loading (1,200,000 cycles, 50 N) + load-to-fracture test (UTM)	3D-printed crowns showed significantly higher fracture resistance than CAD/CAM in all thicknesses; strength increased with thickness	3D-printed crowns demonstrated superior fracture resistance under tested conditions
Henderson et al. [28] (2022)	In vitro experimental	CAD/CAM PMMA vs. 3D-printed bis-acryl vs. conventional bis-acryl	n ≈ 15 per subgroup	Fracture resistance (N)	Storage conditions (1 day vs. 30 days, 37 °C humidity) + load-to-fracture test (Instron)	CAD/CAM showed highest fracture resistance; 3D-printed materials showed lower values and significant degradation over time	CAD/CAM materials exhibit superior mechanical performance; 3D materials affected by aging
Handermann et al. [29] (2024)	In vitro experimental	3D-printed zirconia vs. CAD/CAM zirconia vs. 3D composite	n = 15 per group	Fracture resistance (N)	Adhesive cementation + load-to-fracture test (0.5 mm/min, 45° loading)	3D zirconia showed higher fracture resistance than CAD/CAM zirconia; composite showed lowest values	3D zirconia may outperform CAD/CAM due to better marginal adaptation
Park et al. [30] (2024)	In vitro experimental	CAD/CAM hybrid ceramic vs. two 3D-printed resins	n = 108 (12 per subgroup; thickness: 0.5, 1.0, 1.5 mm)	Fracture resistance (N)	Crown-based testing + load-to-fracture (UTM) + Vickers hardness + SEM analysis	CAD/CAM strength increased with thickness; 3D-printed resins decreased with thickness; highest value observed in 3D resin (P1) at 0.5 mm (3769.7 N)	Mechanical behavior depends on material composition and thickness; all materials exceed occlusal forces

## Data Availability

The original contributions presented in this study are included in the article and Supplementary Material. Further inquiries can be directed to the corresponding author.

## Supplementary Materials

The following supporting information can be downloaded at: https://www.mdpi.com/article/10.3390/dj14060325/s1, File S1:PRISMA 2020 checklist.
